# Oct-4 and Nanog promote the epithelial-mesenchymal transition of breast cancer stem cells and are associated with poor prognosis in breast cancer patients

**DOI:** 10.18632/oncotarget.2506

**Published:** 2014-10-04

**Authors:** Dan Wang, Ping Lu, Hao Zhang, Minna Luo, Xin Zhang, Xiaofei Wei, Jiyue Gao, Zuowei Zhao, Caigang Liu

**Affiliations:** Breast disease and Reconstruction center, Breast cancer key lab of Dalian, the Second Hospital of Dalian Medical University, Dalian, 116023, China

**Keywords:** breast cancer, EMT, Oct-4, prognosis, metastasis, Nanog

## Abstract

Oct-4 and Nanog in regulating the epithelial-mesenchymal transition (EMT) and metastasis of breast cancer has not been clarified. We found that both Oct-4 and Nanog expression were significantly associated with tumor pathology and poor prognosis in 126 breast cancer patients. Characterization of CD44+CD24-Cancer stem cell(CSC) derived from breast cancer cells indicated that CSC rapidly formed mammospheres and had potent tumorigenicity *in vivo*. Furthermore, TGF-β up-regulated the expression of Oct-4, Nanog, N-cadherin, vimentin, Slug, and Snail, but down-regulated E-cadherin and cytokeratin 18 expression, demonstrating that CSC underwent EMT. Knockdown of both Oct-4 and Nanog expression inhibited spontaneous changes in the expression of EMT-related genes, while induction of both Oct-4 and Nanog over-expression enhanced spontaneous changes in the expression of EMT-related genes in CSC. However, perturbing alternation of Oct-4 and Nanog expression also modulated TGF-β-induced EMT-related gene expression in CSC. Induction of Oct-4 and Nanog over-expression enhanced the invasiveness of CSC, but knockdown of both Oct-4 and Nanog inhibited the migration of CSC *in vitro*. Our data suggest that both Oct-4 and Nanog may serve as biomarkers for evaluating breast cancer prognosis. Our findings indicate that Oct-4 and Nanog positively regulate the EMT process, contributing to breast cancer metastasis.

## INTRODUCTION

Breast cancer is the most common malignancy in women; currently, 115 million patients have breast cancer, resulting in 410,000 deaths annually worldwide [1]. Although current anti-tumor therapies have greatly improved the 5-year survival rate of breast cancer patients, recurrence and long-distance metastasis of breast cancer after surgical resection of the primary tumor are often incurable and are the leading causes of mortality in breast cancer patients [2]. Currently, the mechanisms underlying breast cancer metastasis are not fully understood.

Increasing evidence has demonstrated that epithelial cancer cells undergo an epithelial-mesenchymal transition (EMT), which is essential in early-stage tumor metastasis [3]. During the EMT process, epithelial cells lose their cell polarity and cell-cell adhesion and became mesenchymal cells that gain migration and invasion properties. After EMT, epithelial cancer cells lose expression of E-cadherin and epithelial cytokeratin 18 (CK-18), but they express N-cadherin, fibronectin, and vimentin. EMT is positively regulated by the transcription factors Snail and Twist that directly and indirectly repress E-cadherin expression [4]. Furthermore, TGF-β and other growth factors can activate the Wnt/β-catenin, Notch, and MAPK pathways to promote Snail and Slug expression, and the latter can promote cell spreading early in the EMT process. Cancer stem cells (CSC) are crucial for cancer recurrence and metastasis [5]. CSC from mouse or human mammary glands or mammary carcinomas express EMT markers [6], while immortalized mammary epithelial cells express EMT master regulators, such as Snail and Twist, and form mammospheres *in vitro*. Apparently, CSC share some characteristics with epithelial cells that undergo the EMT process. Indeed, the frequency of CSC correlates with the invasive potential of breast cancer cells [7]. In pancreatic cancer, CD133+CXCR4+ pancreatic CSC have EMT characteristics and invasiveness, which can be specifically blocked by a CXCR4 inhibitor [8]. However, it is unclear how the EMT process is regulated in CSC during the development and progression of breast cancer.

Oct-4 is a Pit-Oct-Unc transcription factor family member and is essential for the maintenance of self-renewal in embryonic stem cells [9, 10]. Nanog is a transcription factor, and Nanog and Oct-4 induce the expression of each other [11]: Both proteins play key roles in maintaining the self-renewal capacity and pluripotency of embryonic stem cells and are biomarkers of CSC [9, 12-16]. Previous studies have shown that Oct-4 and Nanog regulate cancer progression [17, 18]. Nanog expression levels are associated with the poor prognosis of colorectal cancer patients [19]. Oct-4 and Nanog co-expression is associated with early stages of pancreatic carcinogenesis [20] and enhanced lung cancer malignancy [21]. However, there is no information on the association of both Oct-4 and Nanog expression with survival of breast cancer patients. There is no information on the impact of simultaneous modulation of Oct-4 and Nanog expression on the EMT process and invasiveness of breast CSC. Therefore, the functional significance of both Oct-4 and Nanog expression in regulating EMT and invasiveness of breast CSC and the prognosis of patients with breast cancer have not been clarified.

In this study, we characterized the levels of Oct-4 and Nanog expression in 126 breast cancer samples and analyzed their association with the clinical pathologic characteristics and prognoses of these patients. Furthermore, we isolated CD44+/CD24− breast CSC from a breast cancer cell line and examined whether simultaneous modulation of both Oct-4 and/or Nanog expression could alter the expression of EMT-related molecules and invasiveness of CSC *in vitro*.

## RESULTS

### Association of Oct-4 and Nanog expression levels with tumor pathology and poor prognosis in breast cancer patients

**Figure 1:**
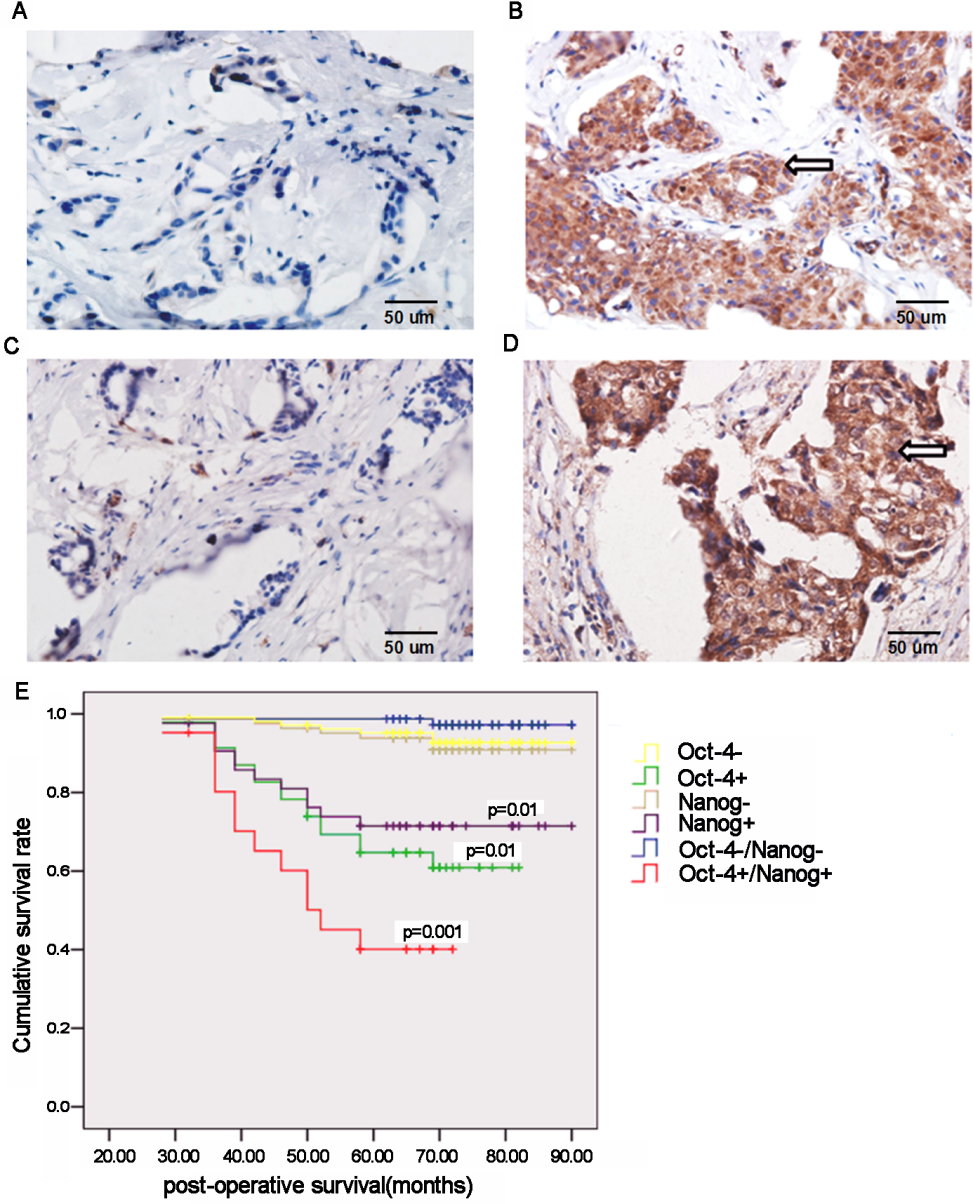
Oct-4 and Nanog expression is associated with the prognosis of breast cancer patients Surgical breast cancer samples were obtained from 126 patients, and the expression of Oct-4 and Nanog in breast cancer tissues and surrounding non-tumor tissues was characterized by immunohistochemistry. Data shown are representative images (magnification ×400). (A) Immunohistochemical analysis of Oct-4 expression in non-tumor tissues. (B) Oct-4 expression in tumor tissues. (C) Immunohistochemical analysis of Nanog expression in non-tumor tissues. (D) Nanog expression in tumor tissues. Arrows indicate positive-staining cells. Subsequently, the patients were stratified based on the expression of Oct-4 and/or Nanog, and their survival was analyzed (E). P values indicate significance, as compared with corresponding groups of patients negative for Oct-4/Nanog expression.

Oct-4 and Nanog are expressed by CSC and are associated with tumor progression [23-25]. 52 (41.27%) out of 126 cases displayed positive staining of anti-Oct-4, 47 (36.51%) cases had positive anti-Nanog staining, and 26 (20%) samples had positive staining for both anti-Oct-4 and anti-Nanog ((Fig. 1A–D), Table 2). Stratification analyses indicated Oct-4 and Nanog expression levels were not associated with patient age or clinical stage of the tumor. However, Oct-4 and Nanog expression were positively associated with tumor size, histological grade, and lymph node status, as well as molecular subtype of breast cancer. Multivariate analysis indicated that lymph node metastasis (odds ratio [OR]: 7.645; 95% confidence interval [CI]: 1.974–29.612; p = 0.003) and the molecular type of breast cancer (OR: 14.776; 95% CI: 3.386–64.477; p = 0.001) were independent factors associated with Oct-4 and Nanog co-expression (Table 3).

Further stratification analyses revealed that cumulative survival in patients doubly positive for anti-Oct-4 and anti-Nanog staining was significantly shorter than that in other groups of patients (p = 0.001; Fig. 1E). Similarly, cumulative survival in patients singly positive for anti-Oct-4 or anti-Nanog staining were significantly shorter than that of patients negative for anti-Oct-4 or anti-Nanog staining (p < 0.01). Therefore, Oct-4 and Nanog co-expression is associated with the poor prognosis of breast cancer patients.

### Characterization of ESA+CD44+CD24-Lin- CSC derived from BT-20 cells

**Figure 2:**
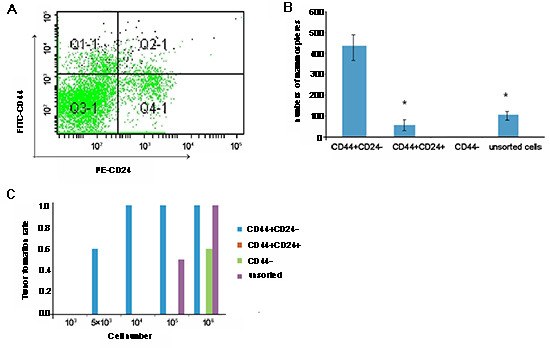
Characterization of cancer stem cells (CSC) from BT-20 cells BT-20 cells were purified from CD44+CD24− breast CSC by flow cytometry sorting by gating CD44+CD24− cells (A) The sorted CSC, together with sorted CD44+CD24+, CD44−, and unsorted tumor cells, were characterized by mammosphere formation assays, and the numbers of mammospheres were counted in a blinded manner (B) Subsequently, the CD44+CD24−, CD44+CD24+, CD44−, and unsorted BT-20 cells were characterized for tumorigenicity in C57BL/6 SCID mice. Briefly, individual SCID mice were implanted with the indicated numbers of each type of cell into their mammary fat pads, and the formation of solid tumors was examined up to 28 days post implantation (C) Data shown are either a representative flow chart, are expressed as the mean ± standard deviation of mammospheres from 5 independent experiments, or are expressed as the ratios of mice with positive tumor formation to those without tumor formation in individual groups (n = 5 per group) following implantation with the indicated numbers of cells. There was no detectable solid tumor following implantation with CD44+CD24+ cells. *p < 0.05 vs. CD44+CD24− CSC.

ESA+CD44+CD24-Lin− CSC were isolated from BT-20 cells by flow cytometry sorting (Fig. 2A). The sorted CD44+CD24− CSC, CD44+CD24+, and unsorted tumor cells, but not CD44− cells, formed typical mammospheres in the presence of leukemia inhibitory factor *in vitro*. Quantitative analysis indicated that the numbers of mammospheres from CD44+CD24− CSC were 5- or 9-fold greater than those from CD44+CD24+ and unsorted tumor cells (*P* < 0.05; Fig. 2B). Subsequently, we tested the tumorigenicity of the sorted CD44^+^CD24−, CD44−, and CD44+CD24+ cells, as well as unmanipulated BT-20 cells, by mammary fat pad injection. Injection of 5 × 10^3^ CD44^+^CD24− CSC induced solid tumors in 3/5 SCID mice, while injection of 10^5^ unmanipulated BT-20 cells triggered the growth of solid tumors in 3/6 SCID mice (Fig. 2C). Injection of 10^6^ CD44−, but not CD44+CD24+, BT-20 cells induced solid tumors in SCID mice. Clearly, CD44+CD24− CSC had stronger tumorigenicity, which may contribute to breast cancer recurrence.

### Simultaneous modulation of Oct-4 and Nanog expression alters the expression of EMT-related genes in CSC

**Figure 3:**
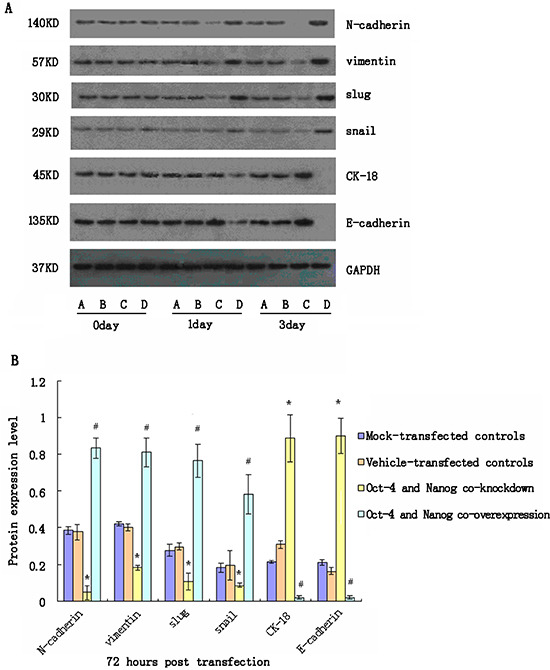
Western blot analyses of relative expression levels of epithelial-mesenchymal transition (EMT)-related genes in cancer stem cells (CSC) following modulating Oct-4 and/or Nanog expression *in vitro* CSC were transfected with mock or Oct-4 and Nanog siRNAs, vehicle, or Oct-4 and Nanog-expressing plasmids for 72 h. The relative expression levels of EMT-related genes in the different groups of cells were characterized at the indicated time points post-stimulation by western blot assays. Data shown are representative images (A) or are expressed as the means ± standard deviation of the relative levels of each protein to the control GAPDH at 72 h post-transfection (B) from 3 separate experiments. A similar pattern of the relative levels of targeting proteins to the control GAPDH were detected in the different groups of CSC at 24 h post-stimulation (data not shown). A: The mock-transfected CSC; B: The vehicle-transfected CSC; C: Oct-4- and Nanog-silenced CSC; D: Oct-4- and Nanog-overexpressing CSC. *p < 0.05 vs. mock-transfected CSC; #p < 0.05 vs. vehicle-transfected CSC.

We further tested the importance of Oct-4 and Nanog co-expression in maintaining EMT characteristics in CSC. We first optimized plasmid transfection conditions for inducing Oct-4 and Nanog over-expression in CSC and screened different siRNAs for knockdown of Oct-4 and Nanog expression in CSC. We found that transfection with Oct-4-specific siRNA2 and Nanog-specific siRNA1 effectively reduced Oct-4 and Nanog mRNA transcription levels by 85–90% at 3 days post-transfection (data not shown). Subsequently, CSC were transfected with mock, vehicle, Oct-4-specific, and Nanog-specific siRNAs, or Oct-4 and Nanog-expressing plasmids, and the relative levels of N-cadherin, vimentin, CK-18, E-cadherin, Slug, and Snail were examined longitudinally by quantitative RT-PCR and western blot assays. We found that simultaneous knockdown of Oct-4 and Nanog expression significantly reduced the relative expression levels of N-cadherin, vimentin, Slug, and Snail, but significantly increased the relative expression levels of E-cadherin and CK-18 in CSC 24 h post-transfection (Fig. 3). In contrast, co-induction of Oct-4 and Nanog over-expression significantly increased expression levels of N-cadherin, vimentin, Slug, and Snail, but decreased expression levels of N-cadherin and CK-18 in CSC (Fig. 3). In addition, a more obvious difference was observed in CSC 72 h post-transfection. Similar patterns of relative mRNA levels were detected in the different groups of CSC at varying time points (data not shown). These 2 separate lines of data demonstrated that Oct-4 and Nanog promote EMT in CSC.

### Perturbing alternation of Oct-4 and Nanog expression modulates TGF-β-induced EMT-related gene expression in CSC

**Figure 4:**
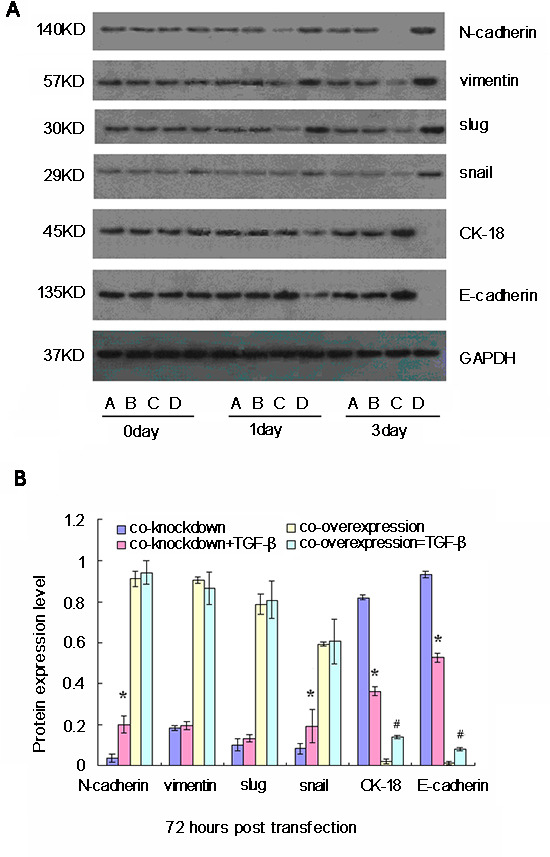
Western blot analyses of relative expression levels of epithelial-mesenchymal transition (EMT)-related genes in cancer stem cells (CSC) following simultaneously modulation of Oct-4 and Nanog expression and TGF-β stimulation *in vitro* CSC were transfected with mock or Oct-4 and Nanog siRNAs, vehicle, or Oct-4 and Nanog-expressing plasmids for 24 h and stimulated with TGF-β for 72 h. The relative expression levels of EMT-related genes in the different groups of cells were characterized at the indicated time points post-stimulation by western blot assays. Data shown are representative images (A) or are expressed as the means ± standard deviation of the relative levels of each protein to the control GAPDH (B) at 72 h post-stimulation from 3 separate experiments. A similar pattern of the relative levels of targeting proteins to the control was detected in the different groups of CSC at 24 h post-stimulation (data not shown). A: Oct-4- and Nanog-silenced CSC; B: TGF-β-stimulated Oct-4- and Nanog-silenced CSC; C: Oct-4- and Nanog-overexpressing CSC; D: TGF-β-stimulated Oct-4- and Nanog-overexpressing CSC. *p   0.05 vs. TGF-β-unstimulated Oct-4- and Nanog-silenced CSC; #p   0.05 vs. TGF-β-unstimulated Oct-4- and Nanog-over-expressing CSC.

To understand the role of Oct-4 and Nanog on TGF-β**−**induced EMT in CSC, we evaluated changes in levels of EMT markers with TGF-β treatment in CSC with either co-silencing or co-overexpression of Oct-4 and Nanog. One day after transfection, regardless of Oct-4/Nanog expression, the relative expression levels of N-cadherin, vimentin, Slug, Snail, E-cadherin, and CK-18 were not significantly changed after TGF-β treatment. At 3 days after transfection, significantly increased levels of N-cadherin were observed in Oct-4- and Nanog-silenced CSC after TGF-β treatment (Fig. 4). Interestingly, treatment with TGF-β did not change the levels of N-cadherin, vimentin, Slug, and Snail, but slightly increased the levels of E-cadherin and CK-18 in the CSC with co-overexpression of Oct-4 and Nanog.

### Modulation of Oct-4 and/or Nanog expression alters the invasion ability of CSC

**Figure 5:**
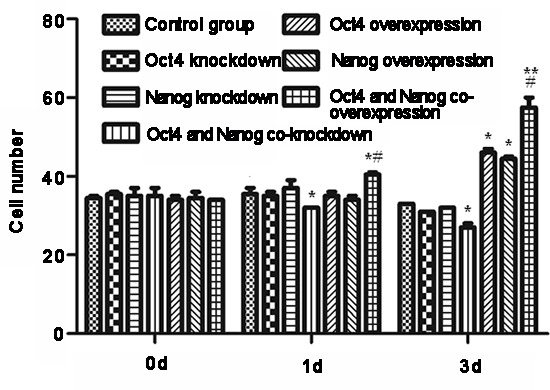
Modulation of Oct-4 and Nanog expression alters the invasiveness of cancer stem cells (CSC) *in vitro* CSC were transfected with Oct-4 and/or Nanog siRNAs or Oct-4 and/or Nanog expressing plasmids for 3 days, and invasive potential of the different groups of cells was examined at the indicated time points post-transfection by transwell migration assays. Data are expressed as means ± standard deviation of numbers of migrated cells in individual groups at each time point from 3 separate experiments. *p < 0.05, **p < 0.01 vs. control and Oct-4- or Nanog-silenced CSC. #p < 0.05 vs. Oct-4- or Nanog-over-expressing CSC.

Finally, we examined the impact of modulating Oct-4 and/or Nanog expression on the invasiveness of CSC *in vitro*. CSC were transfected with Oct-4-specific and/or Nanog-specific siRNA(s) to silence gene expression or with Oct-4 and/or Nanog-expressing plasmids for inducing gene over-expression. Subsequently, the invasiveness of different groups of cells was tested by transwell migration assays (Fig. 5). Although knockdown of either Oct-4 or Nanog did not affect the migration of CSC, simultaneous knockdown of Oct-4/Nanog did reduce the numbers of migrated cells, as compared with vehicle-transfected CSC (p < 0.05). In contrast, induction of either Oct-4 or Nanog over-expression significantly increased the numbers of migrated cells. Over-expression of both Oct-4/Nanog synergistically increased the numbers of migrated cells, as compared with that of Oct-4 or Nanog-over-expressing CSC (p < 0.05). Thus, both Oct-4 and Nanog positively regulate the invasiveness of CSC *in vitro*.

## DISCUSSION

Oct-4 and Nanog are transcription factors and biomarkers for breast CSC [22, 23]. Breast CSC are associated with the relapse and metastasis of breast cancer [24-26]. In this study, we first characterized the relative expression levels of Oct-4 and Nanog in breast cancer and surrounding non-tumor tissues from 126 patients. While there was no detectable Oct-4 and Nanog expression in non-tumor breast tissues, 41.27% of breast cancer samples were positive for Oct-4, 36.51% were positive Nanog, and 20% were positive for both Oct-4 and Nanog. Hence, there is a significant difference in the distribution of Oct-4 and Nanog expression between breast non-tumor and tumor tissues. Furthermore, positive Oct-4 and Nanog expression in breast cancer was not associated with patient age or clinical stage of the tumor, but was significantly associated with tumor size, histological grade, lymph node status, and molecular subtype. Multivariate analysis indicated that Oct-4 and Nanog co-expression was independently associated with lymph node metastasis and the molecular type of breast cancer. More importantly, the co-expression of Oct-4 and Nanog in breast cancer was significantly associated with reduced cumulative survival in breast cancer patients. Our previous studies and those of others have shown that the expression of stemness transcriptional factors in breast cancer correlated with disease stage, and Oct-4 expression is associated with poor disease-specific survival of breast cancer patients [27,28]. Our findings from this study further suggest that Oct-4 and Nanog co-expression may be a valuable biomarker to predict the outcome of patients with breast cancer.

It is well known that EMT of cancer cells is crucial for cancer metastasis [29]. The role of Oct-4 and Nanog expression in the EMT process and metastasis of breast cancer remains controversial [21, 25]. While Oct-4 expression is associated with lymph node metastasis and increased expression levels of EMT markers [30], another study indicated that down-regulation of Oct-4 expression induces EMT in breast cancer cells *in vitro* [31]. In this study, we characterized CD44+CD24- breast CSC from a human breast cancer cell line, BT-20 cells, and found that CSC had potent capacity to form mammospheres *in vitro* and solid tumors in SCID mice, consistent with previous studies [32, 33]. Furthermore, simultaneous knockdown of Oct-4 and Nanog with specific siRNAs significantly enhanced expression levels of E-cadherin and CK-18 and reduced expression levels of N-cadherin, vimentin, Slug, and Snail in CSC. In contrast, induction of both Oct-4 and Nanog over-expression significantly up-regulated expression levels of N-cadherin, vimentin, Slug, and Snail, but almost eliminated expression of E-cadherin and CK-18 in CSC. Increased expression levels of N-cadherin, vimentin, Slug, and Snail and reduced expression levels of E-cadherin and CK-18 demonstrated that over-expression of both Oct-4 and Nanog induced EMT in CSC. The EMT process in CSC is crucial for cancer metastasis. Indeed, induction of either Oct-4 or Nanog over-expression promoted the migration of CSC *in vitro*, and Oct-4 and Nanog co-expression was associated with poor prognosis in patients with lung or liver cancer [21, 34]. Thus, our data may explain the association between Oct-4 and Nanog expression and lymph node metastasis in breast cancer. It is possible that Oct-4 is crucial for the EMT process of CSC but may interfere with the EMT process of non-stem breast cancer cells. We are interested in further investigation of how Oct-4 and Nanog expression promotes the EMT process and metastasis of CSC.

The TGF-β/Smad pathway plays an important role in promoting EMT in tumors. TGF-β binds to its receptors and induces Smad3 phosphorylation. Phosphorylated Smad3 can interact with Snail1 and Twist to form the EMT-promoting Smad complexes that can either repress epithelial genes or activate mesenchymal genes [35]. In this study, we found that TGF-β treatment up-regulated N-cadherin and vimentin expression, but inhibited E-cadherin expression, thereby promoting EMT in CSC *in vitro*. Similarly, TGF-β enhanced N-cadherin and Snail expression, but inhibited E-cadherin and CK-18 expression in the Oct-4- and Nanog-silenced CSC. Indeed, TGF-β treatment increased the proportion of EMT-like cells in CSC. These data suggest that remaining Oct-4 and Nanog may be sufficient to support TGF-β-induced EMT in Oct-4- and Nanog-silenced CSC. Alternatively, TGF-β may induce the EMT process in CSC independently of Oct-4 and Nanog expression. In addition, we found that TGF-β treatment increased the expression levels of Oct-4 and Nanog in EMT-like CSC, similar to non-EMT CSC. These data suggest that TGF-β-activated Smad3 may bind to Oct-4 and Nanog promoters to up-regulate their expression, like a phenomenon observed in embryonic stem cells [36]. Moreover, we found that TGF-β did not significantly alter the expression levels of N-cadherin, vimentin, Slug, and Snail and only slightly enhanced expression of E-cadherin and CK-18 in Oct-4 and Nanog over-expressing CSC. Apparently, Oct-4 and Nanog over-expression may be sufficient to promote and maintain the mesenchymal status of CSC. Given that TGF-β1 can also promote the proliferation of epithelial breast cancer cells, TGF-β1 may enhance non-EMT CSC proliferation, leading to increased levels of E-cadherin and CK-18 in our experimental conditions. Hence, it is possible that TGF-β is a potent inducer of the EMT process in CSC by promoting the expression of EMT-related genes, Oct-4 and Nanog, which may be crucial for maintaining CSC mesenchymal status, thereby promoting the invasiveness and metastasis of breast CSC. Indeed, a previous study has shown that increased levels of plasma TGF-β are associated with poor outcomes in breast cancer patients [24]. Therefore, the TGF-β/Sma3 pathway and Oct-4/Nanog may synergistically promote the invasion and metastasis of breast cancer.

In summary, our data indicated that Oct-4 and Nanog co-expression was associated with lymph node metastasis and the molecular type of breast cancer, as well as poor prognosis in breast cancer patients. Oct-4 and Nanog co-expression may be a valuable biomarker to predict the outcome of breast cancer patients. Furthermore, we found that CD44+CD24− breast CSC had potent capacity to form mammospheres *in vitro* and solid tumors in SCID mice. Oct-4 and Nanog co-expression promoted mesenchymal marker expression and invasiveness of CSC. TGF-β treatment induced expression of Oct-4, Nanog, and mesenchymal markers in CSC, and Oct-4 and Nanog over-expression was sufficient to maintain the mesenchymal status of CSC. Our study had the limitation of a small sample size and lack of experiments in vivo. In addition, there was a lack of functional studies about how Oct-4 and Nanog regulate the EMT and invasiveness of breast CSC. Thus, further investigations with a bigger population are warranted.

## MATERIALS AND METHODS

### Clinical samples

A total of 126 breast cancer samples were obtained from the First Hospital of China Medical University and Liaoning Cancer Hospital and Institute between January 2003 and December 2006. Patients with breast cancer were diagnosed by histological examination of surgical tissue samples, and they underwent radical operations. The inclusion criteria were: (a) undergoing a curative operation, (b) resected tumor specimens were pathologically examined, (c) > 15 lymph nodes were pathologically examined after the operation, and (d) a complete medical record was available. The demographic and clinical data of individual patients were obtained from medical records. Individuals with breast cancer, but not fulfilling the criteria for inclusion, were excluded. Written informed consent was obtained from individual patients, and the experimental protocol was approved by the Ethics Committee of China Medical University.

### Histology and immunohistochemistry

Individual breast cancer tissue samples were fixed in 10% formaldehyde solution (pH 7.0) and paraffin-embedded. The paraffin-embedded tissue sections (4 μm) were stained with hematoxylin and eosin and examined under a light microscope by pathologists in a blinded manner. In addition, Oct-4 and Nanog expression levels were characterized by immunohistochemistry. Briefly, the paraffin-embedded breast tumor tissue sections (4 μm) were de-waxed, rehydrated, and treated with 3% H_2_O_2_ in methanol, followed by incubation overnight with primary antibodies against Oct-4 and Nanog (Abcam, Boston, USA). Subsequently, the sections were incubated with Multilink Swine anti-goat/mouse/rabbit IgG (biotinylated; Dako, Carpinteria, USA). After being washed, the bound antibodies were detected with horseradish peroxidase-conjugated avidin-biotin complex (1:1000 dilutions; Vector Laboratories, Burlingame, USA) and visualized using 3, 3-diaminobenzidine. The sections were then counterstained with Gill's hematoxylin.

The intensity of anti-Oct-4 and anti-Nanog staining was semi-quantitatively analyzed. Individual cells with yellow-to-brown staining in the nucleus and/or cytoplasm of the cells were considered positive cells. Oct-4 and Nanog expression levels were scored semi-quantitatively according to the following criteria: - if < 1% of neoplastic cells expressed Oct-4 and Nanog; + if ≥ 1% of morphologically unequivocal neoplastic cells expressed Oct-4 and Nanog.

### Cell lines

The human breast basal epithelial cancer cell line BT-20 was cultured in Dulbecco's modified Eagles medium containing 10% fetal calf serum (Invitrogen, Grand Island, USA). BT-20-derived CSC were maintained in complete MammoCult™ Medium (10% MammoCult™ Proliferation Supplements [Human] in MammoCult™ Basal Medium; Stem Cell Technologies, San Diego, USA).

### Flow cytometry

Trypsinized BT-20 cells (10^6^/tube) were washed and stained with FITC-anti-CD44, APC-anti-CD24, and 7-aminoactinomycin D for 20 min on ice. After being washed, the dead cells (7-aminoactinomycin D+) were eliminated, and the CD44-CD24−, CD44+CD24− and CD44+CD24+ cells were sorted on a FACS vantage (FACSCALIBUR; BD Biosciences, San Jose, USA). The sorted cells were characterized by flow cytometry, and individual cell samples with a purity of > 95% were used for the following experiments.

### Mammosphere growth and *in vivo* xenograft assays

Aliquots of 5,000 cells of the sorted ESA+CD44+CD24-Lin- breast CSC, CD44+CD24+, CD44−, and unsorted breast cancer cells were seeded into 6-well ultralow attachment plates (Corning, Acton, MA, USA) and cultured in Complete MammoCult™ Medium in the presence of human leukemia inhibitory factor (50 ng/mL), a potent inhibitor of stem cell differentiation, to accelerate the formation of mammospheres. The treated cells were replenished with fresh medium every 3 days and cultured for 7 days. At the end of culturing, the number of spheres with a size of 60 μm or more was counted under a phase-contrast microscope.

Eight-week-old female C57BL/6 SCID mice (Jackson Laboratory, Beijing, China) were housed in a specific pathogen-free facility on our campus. Individual C57BL/6 SCID mice were implanted with different numbers (10^3^–10^6^/mouse) of sorted CD44+CD24−, CD44+CD24+, CD44−, and unsorted tumor cells in 50 μl PBS in their mammary fat pads. The growth of implanted tumors was monitored up to 28 days after xenotransplantation. The protocol for the animal study was approved by the Animal Care and Research Committee of China Medical University.

### Construction and transfection of plasmids for Oct-4 and Nanog over-expression

The cDNA fragments for Oct-4 and Nanog were amplified by PCR using specific primers (Table 1) from a human placenta cDNA library that we constructed previously and cloned into a pEGFP-C1 vector (Clontech, Mountain View, USA) to generate plasmids of pEGFP-Oct-4 and pEGFP-Nanog, respectively, followed by DNA sequencing.

**Table 1:** The sequences of primers

Gene	Primer	Amplicon
Oct-4	Sense: 5′- ggtggaagctgacaaca -3′	159 bp
	Antisense: 5′- atctgctgcagtgtgggttt-3′	
Nanog	Sense: 5′- gtcccaaaggcaaacaaccc -3′	108bp
	Antisense: 5′- gctgggtggaagagaacaca-3′	
Snail	Sense: 5′- ggccttcaactgcaaatact-3′	246bp
	Antisense: 5′- ttgacatctgagtgggtctg-3′	
Slug	Sense: 5′- cttcctggtcaagaagcatt-3′	232bp
	Antisense: 5′- tgaggagtatccggaaagag-3′	
Vimentin	Sense: 5′- tccaagtttgctgacctctc-3′	185bp
	Antisense: 5′-tcaacggcaaagttctcttc-3′	
E-cadherin	Sense: 5′- aacgcattgccacatacac -3′	183bp
	Antisense: 5′- aacgcattgccacatacac -3′	
N-cadherin	Sense: 5′-aactccaggggaccttttc-3′	195bp
	Antisense: 5′-caaatgaaaccgggctatc-3′	
CK-18	Sense: 5′- gggagcacttggagaagaa-3′	195bp
	Antisense: 5′- tggccagctctgtctcata-3′	
β-actin	Sense: 5′- cattaaggagaagctgtgct-3′	208bp
	Antisense: 5′- gttgaaggtagtttcgtgga-3′	

The sorted CD44+CD24− breast CSC were cultured in Complete MammoCult™ Medium on 6-well plates. Upon the cells reaching 70% confluency, the cells were transfected with 2 μg pEGFP-Oct-4, pEGFP-Nanog, control pEGFP-C1, 50 mM Oct-4-siRNA, Nanog-siRNA, or control siRNA (Table 2; Santa Cruz Biotech, Santa Cruz, USA), respectively, using Lipofectamine™ (Invitrogen), according the manufacturer's instruction. The cells were cultured in the presence or absence of 50 ng/ml TGF-β1 and harvested at 1 and 3 days post-transfection for the following experiments.

**Table 2:** The sequences of Oct-4 and Nanog-specific siRNAs

gene	siRNA
Oct-4	siRNA-1: GGTCTCTCTTTCTGTCCTTTC
	siRNA-2: GGGTAGGTTATTTCTAGAAGT
Nanog	siRNA-3: CGTAGGTTCTTGAATCCCGAA
	siRNA-1: GCATCCGACTGTAAAGAATCT
	siRNA-2: CCTGGAACAGTCCCTTCTATA
	siRNA-3: GCAACCAGACCTGGAACAATT

### Transwell invasion assay

The sorted ESA+CD44+CD24-lin- breast CSC were transfected with individual plasmids or siRNA in the presence or absence of 50 ng/ml TGF-β1 for various time periods, and cells (5 × 10^3^cells/well) were cultured in triplicate in the upper chamber of transwell plates (Corning) that had been loaded with 60–80 μl of diluted matrigel (BD Biosciences, San Jose, USA). After incubating for 1.5 h at 37°C, the migrated cells at the bottom of the membrane were fixed with 95% ethanol and visualized using hematoxylin. To quantify the invading cells, 10 random fields (magnification × 400) were selected in each chamber, and the numbers of cells in each field were counted in a blinded manner.

### RNA isolation and quantitative real-time PCR

Total RNA was isolated from different groups of CSC that had been transfected with either a plasmid or siRNA using Trizole (Invitrogen); RNA was reverse transcribed into cDNA using Revert Aid TM first cDNA synthesis kit (Fermentas), according to manufacturer's instruction. The relative levels of vimentin, E-cadherin, N-cadherin, Snail, Slug, and CK-18 mRNA transcripts to the control GAPDH were determined by quantitative real-time PCR using SYBR Green PCR Master Mix and specific primers on Applied Biosystems 7500 Fast Real-Time PCR System. Primer sequences are shown in Table 1. Amplification was performed at 95°C for 5 min, followed by 40 cycles of 94°C for 15s, 55°C for 20s, and 72°C for 20s, followed by extension at 72°C for 7 min. The relative levels of mRNA transcripts were analyzed by 2^−ΔΔCt^.

**Table 3:** Stratification analysis of Oct-4 and Nanog expression in 126 breast cancer samples

Factors	N	Oct^+^	Nan^+^	Nan/Oct^+^	Nan/Oct^−^	P_1_	P_2_	P_3_
**Age**								
< 35 Y	18	4	8	2	8	0.062	0.499	0.281
> 35 Y	108	48	39	24	45			
**Tumor size**	24	8	2	0	14	0.562	0.005	0.016
T1								
T2	83	37	36	20	30			
T3	19	7	9	6	9			
**Histological grade**								
I	15	1	2	0	12	0.001	0.001	0.001
II	57	12	10	4	39			
III	54	39	35	22	2			
**Clinical stage**								
DCIS	16	9	8	1	0	0.151	0.280	0.189
IDC	110	43	39	25	53			
**Lymph node metastasis**								
pN0	50	12	9	2	31	0.006	0.001	0.001
pN1	44	20	15	6	15			
pN2	27	17	19	15	6			
pN3	5	3	4	3	1			
**Molecular type**								
Luminal A	69	20	20	6	35	0.001	0.005	0.001
Luminal B	12	4	4	1	5			
Basal-like	19	15	14	12	2			
Her-2+	26	13	9	7	11			

DCIS, ductal carcinoma in situ; IDC, Invasive ductal carcinoma; **P_1_**: Oct-4; P_**2**_: Nanog; P_**3**_: Oct-4+/Nanog+.

### Western blot analysis

The CSC were collected at different time points after transfection, and total proteins were extracted using a total protein extraction kit (ProMab). After quantification of protein concentrations using a BCA assay (Santa Cruz Biotech), the individual cell lysates (20 μg/lane) were separated by sodium dodecyl sulfate polyacrylamide gel electrophoresis and transferred onto polyvinylidene fluoride membranes. The membranes were blocked with 5% fat-free dry-milk in TBST and incubated with rabbit anti-Slug (1:500; Cell Signaling Technology), mouse anti-vimentin (1:400), mouse anti-GAPDH (1:800), goat anti-E-cadherin (1:500; Santa Cruz Biotech), mouse anti-Snail (1:500; Cell Signaling Technology), rabbit anti-N-cadherin (1:500), and rabbit anti-CK 18 (1:5000; Abcam) at 4°C overnight, respectively. After washing, the bound antibodies were detected with horseradish peroxidase-conjugated anti-rabbit, anti-mouse, or anti-goat IgG at room temperature for 1 h and visualized using enhanced chemiluminescence. The relative levels of targeting proteins to the control GAPDH were determined using ImmuNe software.

### Statistical analysis

All data were analyzed with SPSS statistics software (Version 13.0; Chicago, IL, USA). Continual data were analyzed by Student's *t-*test, and categorical data were analyzed by the chi-square test or Fisher's exact test. Cumulative survival of individual groups of patients was analyzed using the Kaplan-Meier method and tested by the log-rank test. Multivariate analysis was performed using the Cox proportional hazards model. P values less than 0.05 were considered statistically significant.
