# Correction: Oct-4 and Nanog promote the epithelial-mesenchymal transition of breast cancer stem cells and are associated with poor prognosis in breast cancer patients

**DOI:** 10.18632/oncotarget.27791

**Published:** 2021-05-11

**Authors:** Dan Wang, Ping Lu, Hao Zhang, Minna Luo, Xin Zhang, Xiaofei Wei, Jiyue Gao, Zuowei Zhao, Caigang Liu

**Affiliations:** ^1^ Breast disease and Reconstruction center, Breast cancer key lab of Dalian, the Second Hospital of Dalian Medical University, Dalian, 116023, China

**This article has been corrected:** Due to errors during figure assembly, Figure 4A is an accidental duplication of Figure 3A. The corrected Figure 4A, obtained using original data, is shown below. The authors declare that these corrections do not change the results or conclusions of this paper.

Original article: Oncotarget. 2014; 5:10803–10815. 10803-10815. https://doi.org/10.18632/oncotarget.2506

**Figure 4:**
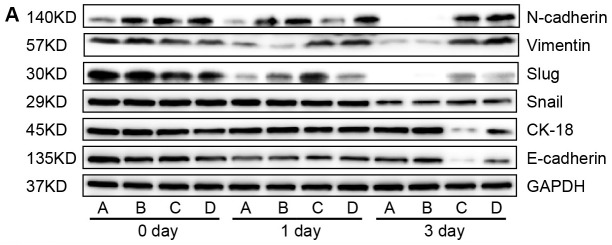
Western blot analyses of relative expression levels of epithelial-mesenchymal transition (EMT)-related genes in cancer stem cells (CSC) following simultaneously modulation of Oct-4 and Nanog expression and TGF-β stimulation *in vitro*. CSC were transfected with mock or Oct-4 and Nanog siRNAs, vehicle, or Oct-4 and Nanog-expressing plasmids for 24 h and stimulated with TGF-β for 72 h. The relative expression levels of EMT-related genes in the different groups of cells were characterized at the indicated time points post-stimulation by western blot assays. Data shown are representative images (**A**) or are expressed as the means ± standard deviation of the relative levels of each protein to the control GAPDH (**B**) at 72 h post-stimulation from 3 separate experiments. A similar pattern of the relative levels of targeting proteins to the control was detected in the different groups of CSC at 24 h post-stimulation (data not shown). A: Oct-4- and Nanog-silenced CSC; B: TGF-β-stimulated Oct-4- and Nanog-silenced CSC; C: Oct-4- and Nanog-overexpressing CSC; D: TGF-β-stimulated Oct-4- and Nanog-overexpressing CSC. ^*^ *p* < 0.05 vs. TGF-β-unstimulated Oct-4- and Nanog-silenced CSC; ^#^ *p* < 0.05 vs. TGF-β-unstimulated Oct-4- and Nanog-over-expressing CSC.

